# Elevated blood lead levels and associated risk factors among school children in a non-industrialized city in Indonesia

**DOI:** 10.1371/journal.pone.0332301

**Published:** 2025-10-08

**Authors:** Nurjannah Nurjannah, Noralina Noralina, Rina Suryani Oktari, Shr-Jie Wang

**Affiliations:** 1 Public Health Department, Medical School, Universitas Syiah Kuala, Banda Aceh, Indonesia; 2 Danish Institute Against Torture (DIGNITY), Copenhagen, Denmark; 3 Magister of Public Health, Medical School, Universitas Syiah Kuala, Banda Aceh, Indonesia; 4 Family Medicine Department, Medical School, Universitas Syiah Kuala, Banda Aceh, Indonesia; Fayetteville State University, UNITED STATES OF AMERICA

## Abstract

Environmental lead exposure in children is a big public health problem in industrial cities in Indonesia. However, exposure to lead can take place in other settings. Our survey in Banda Aceh was the first in a non-industrial Indonesian city. This study was conducted with 130 randomly selected children between October and December 2022, by examining their blood lead samples and administering structured questionnaires on family demographics and environmental factors (i.e., water source and roof type). Following the Centers for Disease Control and Prevention (CDC) guidelines, an elevated blood lead level (BLL) was defined as ≥3.5 μg/dL. Of 130 children enrolled in the study, 32 (24.6%) had elevated BLLs. The mean BLL was 3.01 ± 1.14 μg/dL. Multivariate analysis showed that male students (OR=4.47, 95%CI = 1.44–13.85, *p* = 0.009), who had mothers with lower education (OR=3.85, 95%CI = 1.35–10.95, *p* = 0.011), and who lived in a house with corrugated iron roofs (OR=8.77, 95%CI = 1.03–74.81, *p* = 0.047) were more likely to have elevated BLLs than their counterparts. Age, lower socioeconomic status (welfare recipients), water sources at home, fathers smoking, and whether living in urban or rural areas were not found to be associated with elevated BLLs. In conclusion, this study provides preliminary evidence of elevated BLLs in children living in a non-industrial city, like Banda Aceh, and identifies the potential source of domestic exposure to lead. Further study with a larger sample is required to confirm the findings. There is a need to review the current environmental health and waste management policies in Banda Aceh and develop preventive measures.

## Introduction

Lead is an environmental pollutant that is a major threat to public health [1]. Environmental exposure is recognized as the main cause of increased blood lead levels (BLLs). Lead can be absorbed into the human body through inhalation, ingestion, and skin absorption from many sources, including air, soil, and water [2]. Lead can affect all age groups, but children are the most vulnerable to exposure and long-term effects such as neurological, cognitive, and physical disorders, which can last throughout their lives [3]. Lead exposure can also cause mental health problems and behavioral disorders in children, which could result in violent or criminal behavior [4,5]. Since 2021, the Centers for Disease Control and Prevention (CDC) has defined BLLs ≥ 3.5µg/ dL as the elevated blood lead levels [6].

In 2020, the United Nations Children’s Fund (UNICEF) reported that lead levels of more than 5 micrograms per deciliter (µg/dL) can be found in one child in three, or up to 800 million children worldwide. At present, the children most exposed to and affected by lead are those in Africa, Asia, Central and South America, and Eastern Europe [7]. In most high-income countries, BLLs have fallen drastically since the phasing out of leaded gasoline and leaded paint [8]. On the contrary, in low- and middle-income countries, particularly in their poor communities, BLLs in children remain high. They are exposed to lead from multiple sources, including leaded gasoline and paint, batteries, cigarettes, foodstuffs, cooking and eating utensils, water pipes, household furniture, house roofs, floors, and toys [4,9]. Several ingredients of cosmetics and traditional medicines have also been known to be contaminated by lead, since it was used as a color additive in cosmetics [10,11].

In Indonesia, more than eight million children are estimated to have BLLs above 5 micrograms per deciliter (µg/dL), a threshold that necessitates intervention and makes them vulnerable to lead poisoning. There is no national monitoring system, so the actual number of affected children is unknown. However, studies have shown that exposure to lead can be a serious problem in certain areas [12]. A study in Jakarta in 2003 concluded that 35% of 423 children aged 6–12 had BLLs ≥ 10 µg/dL and 2.4% had BLLs ≥ 20 µg/dL [13]. Recent research in communities near Used Lead-Acid Battery (ULAB) recycling sites, iron mines, or other potential lead sources revealed children’s elevated BLLs. In four communities in Java, 34.9% of 564 children aged 12–60 months had BLLs > 10–20 μg/dL [14], 69.5% of 128 toddlers had BLLs of >10 μg/dL in Bogor, West Java [15], and in Bangka Island around the iron mining areas, 57.5% of 193 children aged 2–9 years old had elevated BLLs (>5 µg/dL) [16].

Factors other than geographical location can play a role. Studies in various places have found a relationship between high lead levels and sociodemographic characteristics such as age, education, and economic status, possibly because they play a role in determining the capacity of parents to provide adequate nutrition and healthcare [11,17–19]. Gender also plays a role; a disparity was found between males and females [20] and there may be differences between urban and rural areas [21].

In Indonesia, detailed data are needed for a variety of areas, including places where there is no obvious source of lead exposure, to enable us to understand the relationship between the presence of environmental lead and elevated blood concentrations. The study presented here was carried out in Banda Aceh, a growing town typical of many areas in Indonesia. For the purpose of this study, the term “non-industrialized city” refers to an urban area without major industrial activities known to emit or use lead extensively, such as battery recycling plants, smelting facilities, or large-scale manufacturing industries. Banda Aceh meets this definition, as it has no major industries directly related to lead production or processing. However, several studies have indicated that residents in Banda Aceh may still be exposed to lead through other pathways, such as contaminated soil in paddy fields close to highways [22], lead contamination in river waters and plants [23,24], and exposure in areas close to the final waste disposal site [25]. It is important to screen BLLs among asymptomatic children, as well as those who show symptoms of lead poisoning, to determine the total number of children who are at risk. This study aimed to measure BLLs among school-aged children in Banda Aceh, a non-industrialized city in Indonesia, and to identify environmental, socioeconomic, behavioral, and geographic risk factors associated with elevated BLLs. Understanding the determinants of elevated BLLs among children in Banda Aceh is pivotal in devising targeted interventions and policy measures aimed at mitigating lead exposure. Such interventions could encompass educational campaigns, stricter environmental regulations, targeted health screenings, and interventions to limit exposure from identified sources.

## Materials and methods

### Study design and participants

A cross-sectional survey was conducted to examine BLLs and their determinants in children aged 12–15 years old in Banda Aceh, the capital city of Aceh Province, located on the northern tip of Sumatra Island, Indonesia (5.5483° N, 95.3238° E). Banda Aceh spans approximately 61.36 km² and has a population of 257,635 in 2022, with an average population density of 4.16 people per km² [26]. The city is classified as urban, but many outer neighborhoods exhibit rural characteristics, with mixed infrastructure, semi-permanent housing, and proximity to agricultural or undeveloped land.

Although Banda Aceh is not home to large-scale, formal industrial activities associated with lead, such as battery recycling, smelting, or manufacturing, it faces anthropogenic sources of lead exposure. These include proximity to highways with heavy traffic, possible use of leaded paints in older homes and schools, and informal practices such as open burning of waste near residential areas. Previous studies have documented lead in paddy soils near highways [22], contaminated river water and aquatic plants [23,24], and elevated lead concentrations near the municipal final waste disposal site [25]. Despite being considered a non-industrialized city by formal classification, these informal or legacy environmental hazards may contribute to children’s lead exposure.

The socioeconomic conditions in Banda Aceh vary across neighborhoods. The provincial poverty rate was estimated at 14.45% in 2022, with adult literacy exceeding 97% [27]. Employment is mixed; while the public sector and trade dominate formal employment, many residents rely on informal labor such as vending, farming, or waste picking [28]. Housing conditions also vary, with some communities having permanent homes and others semi-permanent structures [29], often using corrugated iron roofs.

Regarding infrastructure and public health services, most residents have access to basic sanitation and piped water, although coverage and quality can vary by neighborhood [30]. Healthcare services are provided through a network of primary health centers and hospitals [31], but lead screening is not routinely conducted. Nutritional status among children varies; stunting remains a moderate public health issue in some areas [32], potentially exacerbating vulnerability to lead absorption. The city’s education infrastructure includes public and private schools at the primary and secondary levels [28]. School facilities are generally adequate, although older school buildings may still contain lead-based materials such as paint or piping.

Banda Aceh was selected for this study due to both its representative characteristics of a non-industrialized urban setting in Indonesia and evidence of environmental lead contamination identified in previous studies. It also offers diversity in housing conditions, infrastructure quality, and socioeconomic status, which makes it an ideal site to explore lead exposure risk factors in a low-to middle-income urban context.

For this study, children were randomly selected from two public junior high schools—one located in the city center and one in a rural area to ensure geographic diversity and to capture potential differences in environmental exposure. The minimum required sample size of 128 students was calculated using the Epi Info™ Sample Size Calculator (CDC), applying the “Sample Size for a Proportion” module. The calculation was based on the following assumptions: a total population of 1,585 students in both selected schools, an anticipated prevalence of elevated BLLs of 10% [19], a margin of error of 0.05, and a 95% confidence interval. To account for the finite nature of the population, the sample was adjusted using the finite population correction (FPC). An additional small buffer was added to anticipate minor non-response or data loss, resulting in a final sample of 130 students, proportionally selected by grade level in each school.

Children from two junior high schools were randomly selected between the 1^st^ of October and the 30^th^ of November 2022. These schools were purposively chosen to represent different geographical settings, one located in the city center (urban), and the other in a rural area, ensuring a diverse range of residency locations, enabling us to compare urban and rural environments. From a total population of 1,585 students across both schools, a stratified random sampling technique with proportional allocation was used. The student lists were first stratified by grade level (Grades 7–9) in each school, and then students were randomly selected within each stratum using a random number generator. This approach ensured that the sample was proportionally distributed across grades and schools and helped maintain representativeness of the overall student population. In total, 130 students were selected proportionally by grade level to meet the required sample size while accounting for minor non-response.

A student was considered eligible if she or he was 12–15 years old, was willing to participate in the study, and had parental consent. Students who were eligible but changed their minds were allowed to withdraw from the study. Students who were afraid of needles or blood tests, or were known to have bleeding disorders, were excluded. The class teachers supported the research team by giving participating students an informed consent form for their parents as well as a form for informed assent for themselves. The signed forms were brought back to the class teacher and forwarded to the researchers.

### Blood sampling and data collection

After written informed consent and assent had been obtained from parents and students, a venous blood sample was collected from each student. Before the blood was drawn, participating students were asked to fast for at least eight hours during the night before the blood was withdrawn. Drinking clean water was allowed, but no food until the blood was withdrawn the next morning. (We also examined blood glucose, for a study presented elsewhere). Venous blood sampling was performed by the private Prodia Laboratory® in Banda Aceh, which has been certified by the International Organization for Standardization (ISO) and was recruited as an independent third-party provider. Peripheral venous blood samples were withdrawn and stored in a trace elements tube with sodium heparin. To minimize discomfort and possible side effects, a skilled and experienced laboratory technician performed the sampling using a single-use small needle. No cases of severe side effects necessitating medical treatment were reported. All blood samples were refrigerated immediately upon collection, stored in a 2–8 °C box on-site, and transported to the Prodia Laboratory® for analysis. The blood concentration of lead was measured by LC-tandem mass spectrometry.

Finally, the researchers administered a structured interview questionnaire to identify possible determinants of exposure and possible sources of environmental lead. The questionnaire was developed by the research team based on a synthesis of previously validated instruments used in similar environmental health studies in low- and middle-income countries, with several items adapted to the Indonesian context and tailored to the feasibility of the study [33,34]. Data collection was carried out using a pretested, structured interview questionnaire, translated into Bahasa, which students completed with the assistance of the research team. The instrument was first drafted in English and then translated into Bahasa Indonesia. It was reviewed by three public health experts for content validity, cultural appropriateness, and clarity. The questionnaire consisted of five main sections: (1) Demographics (e.g., age, gender, school, residential address (urban/rural); (2) Parental socioeconomic information (e.g., education level, receive social welfare); (3) Housing characteristics (e.g., roof material); (4) Environmental exposures (e.g., water sources); and (5) Behavioral and health history (e.g., smoking exposure from parents).

Data were collected using online-based questionnaires, administered using Kobo Toolbox through interviewer-assisted completion to ensure consistency and support student understanding. The selected student used the computer laboratory in the school to complete the questionnaire. The trained research team guided each student through the questions to minimize response errors and address any difficulties with language or comprehension.

### Ethics

The study was conducted following the Declaration of Helsinki and approved by the Human Subject Institutional Review Board of Universitas Syiah Kuala, Banda Aceh, Indonesia, under Ethics Approval Letter No. 004/EA/FK/2022.

### Statistical analysis

The dependent variable is BLLs in children, which were categorized into normal and elevated BLLs following the guideline from the CDC that has recommended the reference value of 3.5 µg/dL since 2021 [35]. In this study, BLLs as outcome were classified as <3,5 µg/dL and ≥3,5 µg/dL. The independent variables include age in years, sex, maternal education, socioeconomic status, father’s smoking use within 6 months, living in an urban or rural area, type of roof material, and water sources. Age was modeled as a continuous variable, and the other variables were modeled as categorical variables. Categorical variables were expressed as percentages and analyzed with the χ^2^‐test. Numerical variables (age) were displayed as mean and standard deviation and analyzed with the Mann-Whitney test. Data were first analyzed by a univariate analysis, which involved a frequency distribution test. Bivariate and multivariate logistic regression models were then used to examine the association of socioeconomic and environmental variables with elevated BLLs. Using the enter method, Model 1 was adjusted for sociodemographic and socioeconomic variables, including age, socioeconomic status, and maternal education. In Model 2, environmental factors, including paternal smoking within the past six months, type of roofing, water source, and area of residence, were added to the variables in Model 1 and jointly adjusted. The adjusted odds ratios (AOR) estimate a subject’s likelihood of having an elevated blood lead level, expressed as a probability between 0.0 and 1.0, with p-values of less than 0.05 being considered statistically significant. We conducted both bivariate (unadjusted) and multivariate (adjusted) analyses to examine the possible interaction and confounding. If the effect estimates remained consistent before and after adjusting for relevant covariates, it suggests the absence of confounding in the relationships examined. Cases with missing values on key predictors were excluded from the final regression models. All analyses were carried out with the SPSS software, version 27.0 [36].

## Results

In total, 130 students were enrolled in this study; 57.7% were males and 42.3% were females. More were from urban areas (61.5%) than from rural areas (38.5%). Slightly more than half of mothers (53%) had a low education level, and 17% of families had received social welfare within 12 months of the year preceding the survey. More than 50% of fathers had smoked in the previous six months. Over 80% of children lived in houses that used corrugated iron roofs, and the rest lived in houses with other types of roofs, such as cement, clay tiles, thatched roofs, etc. The household water resources come from a tap of the city water system (73.1%) or a well (26.9%). The mean of BLLs was 3.01 µg/dL (range 1.10–7.60 µg/dL). About 24.6% (n = 32) had elevated BLLs based on the reference value.

Table 1 shows participants’ sociodemographic and environmental determinants in relation to BLLs. Several determinants were not found to be associated with elevated BLLs in our study population. There was no association with living in an urban or rural area; with the domestic water source; with whether or not the family had received social welfare within 12 months, or with the father smoking within the last 6 months. However, statistically significant associations with elevated BLLs were found with age, sex, maternal education, and the type of roof in the participant’s home (*p* = 0.036, < 0.001, 0.001, 0.008, respectively).

**Table 1:** Sociodemographic and Environmental Factors vs. Blood Lead Level Category (n = 130).

Characteristics	Normal blood lead levels		Elevated blood lead levels		Total		p-value^a^
	n	%	n	%	n	%	
Age (years)^*^ mean (SD)	13.14 (0.43)		13.34 (0.60)		13.19 (0.48)		0.036^b^
Sex							
Male	48	49	28	84.4	75	57.7	<0.001
Female	50	51	5	15.6	55	42.3	
Education level of mothers							
Lower	44	44.9	25	78.1	69	53.1	0.001
Higher	54	55.1	7	21.9	61	46.9	
Family receives social welfare within 12 months							
Yes	16	16.3	7	21.9	23	17.7	0.475
No	82	83.7	25	78.1	107	82.3	
Father smoked within 6 months							
Yes	50	51	22	68.8	72	55.4	0.080
No	48	49	10	31.3	58	44.6	
Area of living							
Urban	58	59.2	22	68.8	80	61.5	0.334
Rural	40	40.8	10	31.3	50	38.5	
Type of roof on homes							
Corrugated iron roofs	74	75.5	31	96.9	105	80.8	0.008
Others	24	24.5	1	3.1	25	19.2	
Water Sources							
Tap	73	74.5	22	68.8	95	73.1	0.525
Well	25	25.5	10	31.3	35	26.9	

*Numerical data presented by mean (SD);

^a^Pearson Chi-Square Test (χ^2^) was used to assess the relationship between the BLLs and the sociodemographic and environmental factors;

^b^Mann-Whitney test used for numerical data not normally distributed

**Fig 1:**
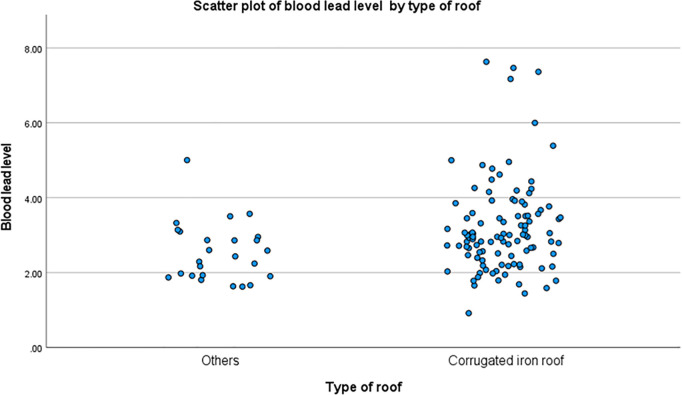
The jitter plots illustrate the BLLs of all study participants based on having a house with a corrugated iron roof or other roof material.

Fig 1 displays the BLL values of the study population against the type of roof construction. When examining the different values of BLLs based on roofing material, the result for the median (interquartile range = IQR) BLL for students living in a home with a corrugated iron roof was 2.9 (1.1–7.6) µg/dL, with a mean value of 3.13. For students whose homes used other materials, the BLL was 2.3 (1.3–4.7) µg/dL, with a mean of 2.51 µg/dL.

Table 2 presents the results of the multivariate logistic regression models. In Model 1, we included sociodemographic and socioeconomic factors, including sex, age, maternal education, and household income level, to examine their association with elevated BLLs. In Model 2, we added environmental variables: father’s smoking status in the past 6 months, type of roof material, water source, and residential area type (urban or rural). In model 1, male students (AOR 4.98, 95% CI: 1.69–14.63*, p = *0.004), who have mothers with lower education (AOR 3.95, 95% CI: 1.46–10.67, *p = *0.007), are likely to have a higher risk of elevated BLLs. The model explained 24.3% of the variance for blood lead levels. In model 2, added environmental factors, being a male student (AOR 4.47, 95%CI: 1.44–13.85, *p = *0.009), and having a mother with lower education (AOR 3.85, 95%CI: 1.35–10.95, *p = *0.011) remained significant and also living in a house with a corrugated iron roof (AOR 8.77, 95%CI: 1.03–74.81, *p = *0.047) were significantly associated with the 8.77 times risk of elevated BLLs. If the father smoked within 6 months, the children were two times more likely to have elevated BLLs, but the result was not statistically significant. Model 2 explained 32.9% of the variance for BLLs. In both Model 1 and Model 2, age was no longer associated with the elevated BLLs. The pseudo-R² values (Nagelkerke R²) were 0.243 for Model 1 and 0.329 for Model 2, indicating improved model explanatory power with the addition of environmental variables. To check for multicollinearity, we examined the variance inflation factor (VIF) for all predictors. All VIF values were below 2.0, indicating no significant multicollinearity.

**Table 2:** Multivariate logistic regression analysis of determinants of blood lead levels (BLLs) in the study population.

Variables	Univariate	Model 1	Model 2
	Odd ratio (95% CI)	Adjusted odds ratio (95% CI)	Adjusted odds ratio (95% CI)
Age (years)	2.23 (1.01–4.91)*	1.29 (0.54–3.10)	1.22 (0.50–2.97)
Sex			
Male	5.62 (2.00–15.81)**	4.98 (1.69–14.63)*	4.47 (1.44–13.85)*
Female	1	1	1
The education level of mothers			
Lower	4.38 (1.73–11.08)**	3.95 (1.46–10.67)*	3.85 (1.35–10.95)**
Higher	1	1	1
Family receives social welfare within 12 months			
Yes	1.43 (0.53–3.88)	0.91 (0.28–2.94)	0.76 (0.22–2.64)
No	1	1	1
Father’s smoking habit within 6 months			
Yes	2.11 (0.91–4.92)		2.02 (0.72–5.3)
No	1		1
Area of living			
Urban	1.52 (0.65–3.55)		1.43 (0.54–3.76)
Rural	1		1
Type of roof			
Corrugated iron roof	10.05 (1.30–77.62)*		8.77 (1.03–74.81)**
Others	1		1
Water sources			
Tap	0.75 (0.31–1.80)		0.71 (0.25–2.03)
Well	1		1
Adjusted R^2^		0.243	0.329

Statistically significant: *(p-value <0.01), **(p-value < 0.05)

## Discussion

To our knowledge, this is the first cross-sectional study in Banda Aceh, Indonesia, to measure BLLs among school-aged children and explore associated environmental and socioeconomic risk factors in a non-industrialized urban setting. We found that around one-quarter of the study population aged 12–15 years have elevated BLLs. The mean BLLs in this study were found to be lower than the values found in previous studies in Indonesia, which were mostly conducted in lead-polluted industrial cities [37,38], likely due to the non-industrial nature of Banda Aceh and the absence of major known sources of lead pollution in the study area. In addition to geographic and environmental differences, factors such as housing characteristics, parental occupation, and waste exposure may also contribute to variations in BLLs. Similar findings of relatively lower BLLs in non-industrial or rural areas have been reported in other parts of the world, such as a study in rural Kenya, where children’s mean BLLs were significantly lower than in urban or mining regions [39], and in the Philippines, where BLLs were notably lower in rural areas of Visayas than in urban or industrial zones [40].

Our study examined sociodemographic factors that could be associated with exposure to lead in the environment. It was found that male students were 4–5 times more likely to have elevated BLLs than female students. This study confirms the findings from the previous studies showing that boys have higher BLLs than girls [20,41,42], though other studies have found no significant difference between mean lead levels in boys and girls [43,44]. Some researchers suggest that this sex-related difference may be influenced by traditional gender roles and boys’ increased participation in outdoor activities, but the exact cause remains unclear [45]. A recent study in China shows that children who play barefoot and frequently put their hands or toys in their mouths are high-risk factors for increased BLLs [20]. It is possible that in Banda Aceh, boys like to play barefoot and do not wash their hands as frequently as girls.

BLLs may also be related to factors other than direct environmental exposure. For example, lead is bound to erythrocytes, which tend to be present in a higher concentration in the blood of the male population. Physiological factors specific to females, such as menstruation, pregnancy, and menopause, as well as hereditary factors, may affect blood lead concentration [46]. This study did not examine the participants’ executive functions, including memory and attention, but another study has shown that these can be detrimentally affected by high BLLs, and this may be especially important for boys, as the effects of lead on girls have been found to be much less [47].

Previous studies showed that socioeconomic status (SES) influenced BLLs directly and indirectly [17,38]. Having less educated parents and a lower income were risk factors associated with higher BLLs in children [15,17,19]. It is suggested that in households with a lower SES, mothers (who are traditionally the primary caregivers in Indonesian culture, which has an impact on the health and education of children) may lack knowledge of the various sources of lead in the environment and ways to prevent exposure. They may not have appropriate housing and may lack access to health services [17,48]. In our study, we considered two SES factors: the level of education of mothers and social welfare status within 12 months in our data analysis. A family with a low-income level is defined as one that receives social welfare based on the criteria defined by the Ministry of Social Affairs, Republic of Indonesia [49]. Our study did not find that receiving social welfare within 12 months was a significant risk factor in determining raised BLLs in the study population. However, we did find that having a mother with a low educational level was statistically significantly associated with elevated BLLs in her children.

As a significant cause of environmental toxicity to public health, lead exposure comes from various potential sources, both indoor and outdoor (soil, leaded gasoline, lead-based paint, leaded pipe for drinking water, contaminated home materials, and food). There is detailed data on exposure to lead in industrialized countries, where the primary sources of exposure for children are contaminated soil, water, paint, and contaminated dust in their homes [50]. Our study emphasizes the prevalence of elevated BLLs and potential domestic sources, including smoking (since lead is one of the elements in tobacco), the type of roof, and the water sources. Although in this present study, the connection between elevated BLLs and the participants’ fathers being smokers was not statistically significant, for the 32 children with elevated BLLs, 22 of them had fathers who had smoked within 6 months, and the risk was two times higher than for the children whose fathers did not smoke. The low statistical power is probably due to the small sample size and the fact that we did not have detailed information on the fathers’ smoking habits (e.g., whether fathers smoke indoors or outdoors and how frequently they smoke at home). A further study with a larger study population and collection of more detailed information is needed. Earlier studies revealed that lead concentration was higher in the blood of smokers than in non-smokers [15,51,52], and children aged 4–16 years who are passive smokers potentially had increased BLLs [53]. Children with at least one smoking parent had a higher probability of BLLs than those with non-smoking parents, which can be worse when fathers habitually smoked inside the house [54].

The majority of the study population lived in houses with corrugated iron roofs. This type of roof was significantly associated with elevated BLLs in children. A corrugated iron roof is a roofing structure composed of metallic materials, including zinc, iron, or steel. This material is frequently utilized due to its availability, cost-effectiveness, strength, durability, and ease of installation. Lead was soldered over holes in corrugated roofs as a traditional patching material [55]. Also, some old houses may use lead-containing paint for coating the corrugated iron roofs. In urban areas, stormwater can leach a significant amount of metal roofing materials, and lead may be found in dust and gutters. A study in a rural area in Bangladesh showed that if the corrugated iron roof was exposed to an acidic solution, for example, acid rainwater, lead could be released [56]. A study in Melbourne also discovered lead levels exceeding the safe limit in rainwater tanks [57]. Our findings suggest that the use of corrugated iron roofs may be associated with elevated BLLs among children, possibly due to lead-containing paint or solder used in patching roof holes. Lead may also leach from roof surfaces into soil or water as a result of acid rain. However, these explanations remain speculative, as this study did not include laboratory testing of roofing materials or environmental samples to confirm the presence of lead. The absence of direct environmental sampling represents a limitation in fully establishing the exposure pathway. Since corrugated iron roofs are used on most houses in Banda Aceh, sampling of roofing materials is recommended, along with collecting additional information on rooftop rainwater harvesting systems, which direct water from a sloped roof into gutters and downspouts and into a storage tank. Future studies should incorporate comprehensive environmental assessments, including testing roof paint, solder residues, and harvested rainwater, to more accurately determine the contribution of specific sources, such as roofing materials and water systems, to lead exposure in non-industrial settings.

In our study, we recorded the students living in the Aceh Besar district or on the border between the Banda Aceh municipality and Aceh Besar as living in a rural area. The study found no significant relation between elevated BLLs in children from urban or rural areas. This is probably because neither area is exposed to industrial pollution, and the distance between the borders of the district is only 10 km. In studies in other places, a marked difference is often found in possible exposure to lead; in rural areas, lead can be present in water and food sources [21,56], and there are major risks of lead contamination in urban areas in air, soil, and water, due to heavier traffic, pollution from large-scale industry, and waste [58]. Higher levels of BLLs were found in Indonesia, particularly in children who lived close to lead smelters or certain industrial sources of lead, such as mining or ULAB recycling sites [13–15]. Water supplies can be important conduits of lead exposure. In several countries, corroded plumbing has been recognized as delivering lead to the human body through drinking water [10,59,60]. Lead might also occur in water from wells that have been contaminated by lead in the soil [61].

This study is the first cross-sectional study to report elevated BLLs among school-aged children in Banda Aceh, a non-industrialized city in Indonesia. One of the strengths of this study is the use of random sample selection from two public junior high schools in both urban and rural areas, which increases the representativeness and generalizability of findings for children in the same age group across the city. Additionally, standardized procedures for blood sample collection and analysis, conducted by an accredited third-party laboratory, ensured the reliability of the BLL measurements.

However, this study also has several limitations. First, this study covered a relatively small number of participants, which may limit the statistical power to detect weaker associations or interactions among variables. Second, although proxy indicators such as roofing material and water source were assessed, the study did not directly measure the presence of lead in different potential sources, such as soil, dust, water, or building materials, which limits our ability to confirm the specific exposure pathways. Third, while elevated BLLs are more commonly observed in younger children, this study focused on adolescents aged 12–15 years, which may underestimate the full extent of lead exposure in the broader child population.

To minimize these limitations, we used a pretested and structured questionnaire, selected participants randomly to reduce selection bias, and ensured ethical and procedural rigor in biological sample collection and handling. Despite these constraints, the study contributes valuable baseline data for public health surveillance and highlights the need for environmental lead monitoring and policy interventions in non-industrial urban settings like Banda Aceh.

## Conclusion

In this study, one-quarter of a sample of students aged 12–15 years in Banda Aceh were found to have elevated BLLs. This was found to be associated with males, children of mothers with a low educational level, and homes with corrugated iron roofs. Other socioeconomic and demographic factors, such as parental smoking and water sources, were not statistically significant in this study, but they should not be overlooked. This study offers the first evidence of elevated BLLs in children living in Indonesia in an area without apparent industrial lead exposure. Further research with a larger sample is needed to confirm and extend these findings. Preventive measures are critical, and follow-up examinations for students with elevated BLLs are necessary. Additional funding has already been secured for a follow-up study to assess whether BLLs remain elevated and if any complications have arisen from prolonged lead exposure.

## Supporting information

S1 File Inclusivity in global research questionnaire. (DOCX)

S1 Data Dataset PONE-D-25-19957. (XLSX)
