# Supplementary material for: Elevated blood lead levels and associated risk factors among school children in a non-industrialized city in Indonesia
Source: PLoS One. 2025 Oct 8;20(10):e0332301. doi: 10.1371/journal.pone.0332301 (PMC12507209; doi:10.1371/journal.pone.0332301)
Supplement: S1 File — (DOCX) [file pone.0332301.s001.docx]

Inclusivity in global research

PLOS’ policy on inclusivity in global research aims to improve transparency in the reporting of research performed outside of researchers’ own country or community and ensures that PLOS publications reporting global research adhere to high standards for research ethics and authorship. Authors of relevant research articles may be asked to complete the questionnaire below, which outlines ethical, cultural, and scientific considerations specific to inclusivity in global research. This questionnaire may be requested when researchers have travelled to a different country to conduct research, if research uses samples collected in another country, research with Indigenous populations or their lands, or if research is on cultural artefacts. Researchers travelling to another country solely to use laboratory equipment will not normally be required to complete the questionnaire. However, the questionnaire can be requested at the journal’s discretion for any submission – if you have been requested to complete this questionnaire by the PLOS journal you submitted to, please do so.

Please complete the questionnaire below and include this as a Supporting Information file with your manuscript. Note that if your paper is accepted for publication, this checklist will be published with your article in the supporting information files. Please ensure that you reference the checklist in the main body of your manuscript. We suggest adding a subsection ‘Inclusivity in global research’ to your Methods section and adding the following sentence: “Additional information regarding the ethical, cultural, and scientific considerations specific to inclusivity in global research is included in the Supporting Information (SX Checklist)”

The questions have been designed to be applicable to a wide range of study types, and there are subsections for both human subjects research and non-human subjects research. If any of the questions are not relevant to your research please mark them as “N/A” as appropriate.

**Ethical considerations, permits and authorship**

*This section is applicable to all research types.*

Provide details as to who granted permissions and/or consent for the study to take place in the Methods section of your manuscript. This should include the names of **all** ethics boards, governmental organizations, community leaders or other bodies that provided approval for the study. If individuals provided approval refer to these people by their role or title but do not list their name(s).

Reported on page number: 214 - 217

If there were any deviations from the study protocol after approval was obtained please provide details of these changes in the Methods section of your manuscript.
Did this study involve local collaborators that are residents of the country where the research was conducted or members of the community studied? If you do not have any authors from said communities, please provide an explanation for this below.

Reported on page number: Not relevant

| Yes, this study involved local collaborators (Nurjannah Nurjannah, Rina Suryani Oktari, Noralina Noralina, Haiyun Nisa) who are residents of the country where the research was conducted and are members of the community studied. All individuals who made substantial contributions to the design, data collection, analysis, and interpretation of the study have been included as co-authors following PLOS’s authorship criteria and listed under the section of “Author Contributions” |
| --- |

Everyone listed as an author should meet PLOS’ criteria for authorship and all individuals who meet these criteria should be included in the author byline, rather than the acknowledgements. For further information please see the journal’s Authorship Policy.

**Human subjects research (e.g. health research, medical research, cross-cultural psychology)**

Did you obtain written informed consent from a representative of the local community or region before the research took place? How did you establish who speaks for the community? Details of written informed consent obtained from study participants should be reported separately in the Methods section of your manuscript.

| Yes, written informed consent was obtained from a recognized representative of the local community (District Education Office of Banda Aceh Municipality) before the research commenced. The head of the District Education Office of Banda Aceh Municipality permitted us to conduct a study in two middle schools in Banda Aceh, called Middle School 17 and Middle School 6. Community representation was established in consultation, including school principals, representatives of the parent-teacher committee, and representatives from the District Education Office that have formal and cultural legitimacy in the region. Informed consent from individual participants was separately documented and is described in the Methods section of the manuscript. Since the study also involved school students, informed assent was obtained from the students in addition to parental consent. Participation was only permitted when both the student and their parent or guardian provided consent and assent. If either party declined, the student was not included in the study. |
| --- |

How did members of the local community provide input on the aims of the research investigation, its methodology, and its anticipated outcome(s)?

| Community members, including school representatives, local health officials (the primary health care center that covers the schools), and institutional stakeholders (District Education Office), were actively engaged in the early stages of the research through consultations and participatory discussions. Their insights informed the formulation of research objectives and the selection of culturally appropriate methods for engaging with school children and their families. This collaborative approach helped ensure the study was contextually relevant, ethically grounded, and sensitive to local health concerns and community norms. For example, in organizing blood drawn, inviting parents and explaining the topic of the blood lead effect on health, and measures in preventing and treating the participants who had elevated blood lead levels. |
| --- |

When engaging with the local community, how did you ensure that the informed consent documents and other materials could be understood by local stakeholders?

| All consent forms and research materials were translated into the local language (Bahasa Indonesia) and reviewed for cultural appropriateness. Trained local facilitators supported the consent process to ensure that participants and stakeholders fully understood the purpose and procedures of the study. |
| --- |

Will the findings of the research be made available in an understandable format to stakeholders in the community where the study was conducted (e.g. via a presentation, summary report, copies of publications, etc.)? Please provide details of how this will be achieved.

| We are committed to sharing the findings with the community in an accessible format. A summary report in the local language has been shared with the District Education Office, school principals, and teachers. Also, we conducted meetings with parents and children to inform their results and what the next steps can be done, either to prevent or treat the elevated blood lead levels. Additionally, we have presented the results at the conference at Universitas Syiah Kuala in 2024. We also plan to share copies of the published article with local partners and institutions after the article has been published. |
| --- |

**Non-human subjects research using specimens/ animals collected as part of the study, or those housed in archival collections. Examples include archaeology, paleontology, botany and zoology.**

Did the permission you obtained from a local authority to perform the study include an agreement on access to outputs and benefit sharing? This may include procedures to enable fair distribution of the benefits and resources arising from the research performed. Please include any details of Prior Informed Consent and Benefit Sharing Agreements obtained. These may be required by field-specific regulations, for example the Convention on Biological Diversity (CBD) and the associated Nagoya Protocol.

| Not relevant |
| --- |

If the material used in your study was imported, please A) provide the year it was imported and B) indicate whether permits were obtained to import/export the materials used, C) provide details of any permits obtained. If this information is not available, please indicate this.

| Not relevant |
| --- |

If you used archival specimens, please state how the material used in your study was acquired by the institute it is held in and provide details of any permits obtained for the original excavations/ sample collection. If this information is not available, please indicate this.

| Not relevant |
| --- |

How was the potential cultural significance of the materials collected in your study to local communities considered in your research design? Were Indigenous peoples and/or local researchers and institutions involved with archaeological excavations / collection of specimens? If so, please provide a description of their involvement.

| Not relevant |
| --- |

If your manuscript includes photographs of human remains please indicate whether authors obtained permission from descendants or affiliated cultural communities to do so.

| Not relevant |
| --- |
